# Singapore grouper iridovirus protein VP088 is essential for viral infectivity

**DOI:** 10.1038/srep31170

**Published:** 2016-08-08

**Authors:** Yongming Yuan, Yunzhi Wang, Qizhi Liu, Feng Zhu, Yunhan Hong

**Affiliations:** 1 Department of Biological Sciences, National University of Singapore, Science Drive 4, Singapore 117543, Singapore

## Abstract

Viral infection is a great challenge in healthcare and agriculture. The Singapore grouper iridovirus (SGIV) is highly infectious to numerous marine fishes and increasingly threatens mariculture and wildlife conservation. SGIV intervention is not available because little is known about key players and their precise roles in SGVI infection. Here we report the precise role of VP088 as a key player in SGIV infection. VP088 was verified as an envelope protein encoded by late gene *orf088*. We show that SGIV could be neutralized with an antibody against VP088. Depletion or deletion of VP088 significantly suppresses SGIV infection without altering viral gene expression and host responses. By precisely quantifying the genome copy numbers of host cells and virions, we reveal that VP088 deletion dramatically reduces SGIV infectivity through inhibiting virus entry without altering viral pathogenicity, genome stability and replication and progeny virus release. These results pinpoint that VP088 is a key player in SGIV entry and represents an ideal target for SGIV intervention.

Viral infection is a great challenge in human healthcare, and it causes regional or global outbreaks of severe human diseases such as influenza, acquired immune deficiency syndrome (AIDS) and currently Zika fever. Viral infection also threatens wildlife conservation and agriculture. For example, iridoviruses infect a wide range of aquatic vertebrates including fish, amphibians and reptiles^1^,^2^, and the Singapore grouper iridovirus (SGIV) can cause massive or even complete loss of wild and farmed groupers as well as many other marine teleosts in aquaculture^3^,^4^. Mechanistic understanding of viral infection is essential for anti-viral approaches in human healthcare, wildlife conservation and agriculture.

SGIV is identified as a novel marine fish DNA virus belonging to genus *Ranavirus*, family *Iridovirdae*^5^. It is highly infectious and causes massive death in groupers and many other marine teleosts^4^,^5^. The SGIV genome is a circular dsDNA of 140,131 bp and predicts 162 open reading frames^6^, which are classified as immediate-early (IE), delayed early (DE) and late (L) genes^2^,^7^. To date, 6 L genes have been studied in SGIV. For instance, *orf088* encodes a putative envelope protein^8^, *orf018* is involved in serine/threonine phosphorylation and virus assembly^9^, *orf019* and *orf038* encode proteins that accumulate in a gathering point with concentrated DNA and proteins for virion assembly, which is termed viral assembly site (VAS). The VAS is also known as the “viral factory” is a dynamic cellular structure that forms late during the viral infection cycle and functions in the key process of viral replication and/or assembly, and thus represents a target for intervention of viral infection^2^,^10^. The VAS of SGIV is a unique subcellular component containing DNA and protein for virus assembly. This DNA gathering point locates at the perinuclear region of the host cell, which can be recognized with DNA staining^11^,^12^. In the VAS, the protein encoded by *orf019* and *orf038* are finally assembled as envelope protein and capsid proteins respectively^12^,^13^. Most recently, late gene *orf039* has been reported to encode envelope protein as a target in SGIV neutralization^14^, and *orf075* has been found to encode a scaffold protein essential for virion packaging and structural stability^15^. However, key players and their precise roles in SGIV infectivity and pathogenicity have remained to be identified.

We are using haploid ES cell HX1 from Medaka fish (*Oryzias latipes*) as host to identify the major viral genes facilitating pathogen infection and propagation. This fish is an excellent vertebrate model for developmental genetics^16^, stem cell biology^17^,^18^,^19^,^20^, reproductive technologies^17^,^20^, gene targeting *in vitro*^21^ and *in vivo*^22^,^23^. Most importantly, HX1 is susceptible to infection by SGIV, spring viremia of carp virus plus nervous necrosis virus^24^ and suitable for the genetic screening for proviral host factors by genome-wide insertional mutagenesis^16^,^25^. Here we identify the SGIV envelope protein VP088 as a key player in viral infectivity. We show that VP088 deletion quantitatively affects SGIV entry without altering viral pathogenicity, genome replication, and the release of progeny virus. Our results precisely pinpoint the primary role of VP088 and make it an ideal candidate for SGIV intervention.

## Results

### VP088 is an envelope protein

**Figure 1:**
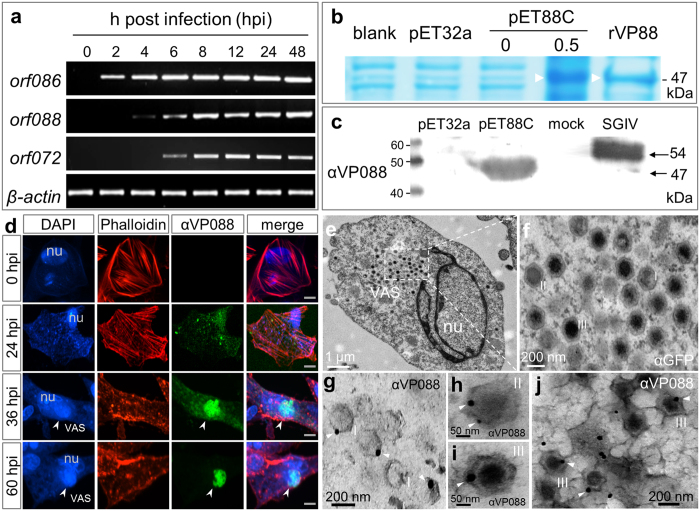
VP088 expression and localization. (**a**) Late transcription of *orf088* in HX1 cells. Early gene *orf086* and late gene *orf072* were used for comparison, and *β-actin* was used as internal control. (**b**) SDS-PAGE, showing expression and purification of recombinant VP088 (rVP88; arrowhead). BL21 bacteria were transformed with vehicle pET32a or VP088-expressing pET88C and induced for expression with IPTG at 0.5 mM, and rVP088 was purified for detection. Lysate from BL21 bacteria was loaded as a negative control (blank). (**c**) Western blot analysis, showing the specificity of αVP088 to detect VP088 expressed in BL21 bacteria and SGIV-infected HX1 cells. (**d**) Confocal microscopic observation of Immunofluorescence from SGIV-infected HX1 cells, showing expression and subcellular distribution of phalloidin-stained F-actin (red) and αVP088-stained VP088 (green). nu, nucleus; VAS, viral assembly site. Scale bars, 5 μm. (**e–j**) Localization of VP088 by immunoelectron microscopy. Ultrathin-sectioned cells at 60 hpi with SGIV were stained with αGFP as control or αVP088 to locate the VP088 with immuno-gold. (**e**) Immunoelectron micrographs of SGIV-infected cells stained with αGFP. (**f**) Higher magnification of boxed area in (**e**), showing negative result of immuno-gold labelling. Meanwhile, SGIV capsids at different assembly stages including empty (I), partial assembly (II) and full assembly (III) were detected in VAS. (**g–j**) Immunoelectron micrographs of SGIV-infected cells stained with αVP088, showing VP088 (immuno-nanogold particles, arrowheads) assembled on the empty capsids (**g**), partial assembled capsid (**h**), fully assembled capsids (**i**) in the VAS and virions outside of VAS (**j**).

VP088 has been reported as a putative envelope protein encoded by late gene *orf088*^8^. An RT-PCR analysis revealed that the transcripts of either *orf088* or well-studied late gene *orf072* were hardly detectable in HX1 cells until 6 hours post infection (hpi), whereas the RNA of immediate early gene *orf086* was apparent as early as 2 hpi (Fig. 1a), which verifies *orf088* as a late gene. To examine VP088 protein expression and localization, we generated a mouse monoclonal antibody αVP088. And a recombinant protein rVP88 was expressed to test the binding ability of αVP088 with West blot analysis. On SDS-PAGE, rVP88 exhibited a molecular weight of 47 kDa as predicted for a protein of 428 amino acid residues including 248 amino acids of truncated protein at the C-terminus of VP088 and additional 180 amino acids of fused tags encoded by pET32a (Fig. 1b). With Western blot analysis, αVP088 clearly detected the purified rVP88, and it also detected a protein with a molecule weight of approximately 54 kDa in the cell lysate from SGIV-infected HX1 cells, which was absent in mock-infected HX1 cells (Fig. 1c). The predicted size of VP088 is 54-kDa according to the amino acids sequence translated from SGIV genome, and the size of protein detected by αVP088 is similar to that of VP088 in the previous report^8^, suggesting that the αVP088 can detect the VP088 specifically. Taken together, VP088 is the product of late gene *orf088*, and αVP088 has specificity to VP088 for detection and interaction.

Subsequently, αVP088 was used for immunofluorescence staining of VP088 in SGIV-infected HX1 cells. Subcellular distribution of VP088 changed along with the intervals after infection (Fig. 1d). At the beginning of infection, a well-organized filament actin (F-actin) network was visible in host cells when VP088 expression was not detectable. However, at 24 hpi, VP088 was clearly detected in the cytoplasm as small granules, and it further condensed at 36 hpi. VP088 protein was highly enriched and colocalized with the concentrated DNA in VAS until 60 hpi (Fig. 1d). To further verify if the VP088 is a structure protein of SGIV, we analyzed the location of VP088 with immunoelectron microscopy. Under electron microscopy, the VAS was detected besides the nucleus of the host cell (Fig. 1e). A closer inspection clearly revealed the presence of empty capsid (I), partially (II) and fully assembled virions (III) in the VAS (Fig. 1e). An antibody against GFP protein (αGFP, ab1218; Abcam) was used as the irrelevant primary antibody. But it cannot detect the target protein in the host cell concluded from the clear background with no or fewer gold particles on the section (Fig. 1f). When the αVP088 was used as a primary antibody, the gold particles precisely indicated the VP088 was located on the shell layer of the empty capsid (Fig. 1g), partially (Fig. 1h) and fully assembled virions (Fig. 1i) in the VAS, and the VP088 was also detected on the matured virions (Fig. 1j) outside of the VAS. Thus, VP088 is located on SGIV capsid as an envelope protein.

### VP088 is essential for viral titer

**Figure 2:**
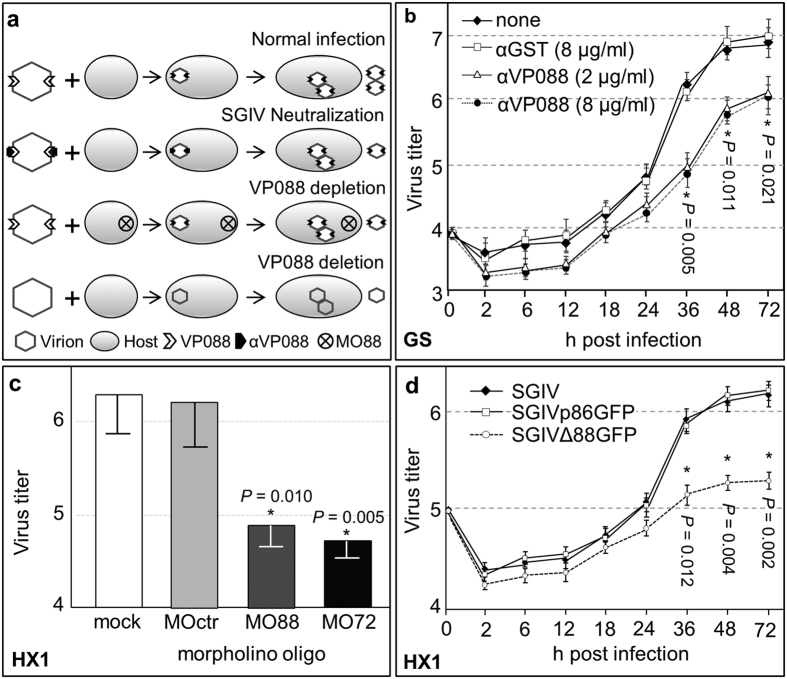
VP088 is essential for virus titer. (**a**) Strategies for identifying key players in SGIV infection, showing normal SGIV infection and its intervention via SGIV neutralization by its antibody αVP088, VP088 depletion by using morpholino (MO88) for gene knockdown and deletion by gene knockout. (**b**) Virus titer of VP088-neutralized SGIV in GS cells. (**c**) Virus titer upon VP088 depletion in HX1 cells at 72 hpi. (**d**) Virus titer upon VP088 deletion in HX1 cells. Virus titers determined at 72 hpi are presented as means ± S.D. (error bars) from three independent experiments. Virus titers were determined as [log_10_ (TCID_50_ ml^−1^)]. *significant difference at *P* < 0.05 compared to control.

The observations that VP088 is an envelope protein provoked us to test VP088 as a potential target for virus intervention. To this end, we employed three approaches, namely SGIV neutralization, *orf088* gene knockdown and gene deletion (Fig. 2a). For virus neutralization, SGIV was pre-incubated with αVP088 before inoculation into GS cells. A time course analysis revealed a decrease in virus titer at all intervals examined, which became significant from 36 hpi onwards when SGIV was neutralized by incubation with 2~8 μg/ml of αVP088 prior to inoculation, whereas pre-incubation with αGST as an irrelevant antibody had no effect on viral titer (Fig. 2b). Clearly, SGIV neutralization with an antibody against VP088 significantly reduced the virus titer.

Three antisense morpholino oligos (MO) were used for gene knockdown experiments. MOctr served a negative control morpholino that has no target in sequenced genomes of SGIV and medaka fish, MO72 served a positive control by targeting major capsid protein encoding gene *orf072*, and MO88 was used to deplete VP088 expression by targeting its encoding gene *orf088*. MO-treated HX1 cells were examined for VP088 expression by Western blot analysis at 72 hpi with SGIV. The level of VP088 was obviously decreased by MO88 or MO72 but not MOctr (Fig. S1). Concomitantly, the viral titer was severely reduced by up to 10 folds via MO88 or MO72 but not MOctr (Fig. 2c). Taken together, VP088 depletion remarkably affects the SGIV titer.

**Figure 3:**
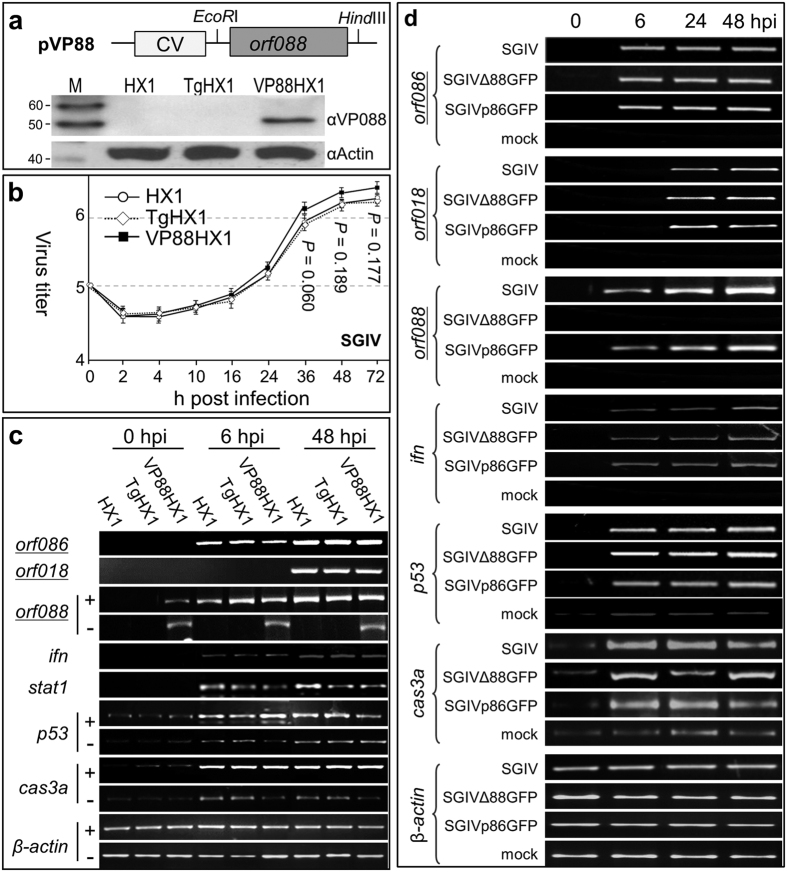
VP088 is dispensable for SGIV gene expression and host response. (**a**) Transgenic HX1 cells VP88HX1 and TgHX1, showing a schematic map of vector pVP88 (top) and Western blot analysis of transgenic VP088 expression in VP088HX1 but not in control cell TgHX1. CV, human cytomegalovirus enhancer/promoter; *orf088*, gene encoding VP088; EcoRI and HindIII, restriction sites used for cloning. (**b**) Virus growth curves. (**c,d**) RT-PCR analysis, showing similar expression patterns of viral genes (underlined) and cellular genes upon VP088 overexpression (VP88HX1; (**c**)) and deletion (SGIV∆88GFP; (**d**)). Plus and minus indicate SGIV infection or mock infection, and negative results for viral genes and immune-response genes are not shown in (**c**). *β–actin* served an internal control.

Finally, we analyzed gene deletion phenotype by the gene knockout technology. Two versions of recombinant SGIV were generated. One is SGIVΔ88GFP, in which the VP088-encoding gene *orf088* was deleted by homologous recombination and replaced by e*gfp* (Fig. S2a). After pBKO88GFP transfection and SGIV infection, homologous recombination occurred in host cell between plasmid DNA and SGIV genome by replacing gene *orf088* with *gfp*. This recombinant genome was packed into progeny virion after assembly. This chimeric virion contains recombinant genome without *orf088* gene, but the VP088 protein encoded by wild-type SGIV was still assembled on its capsid. The chimeric virions and wild-type SGIV were released from the host for the next life cycle, and both of them should have the same infectivity. In the second run of infection, the recombinant genome carried by the chimeric virus was subsequently replicated for progeny virion assembly. After the second run of infection, the amount of chimeric virus increased. Meanwhile, the *gfp* gene in the recombinant genome was expressed in host cells. However, the GFP signal in host cells doesn’t guarantee that the chimeric virus was generated, since the *gfp* gene in plasmid pBKO88GFP may also be expressed in SGIV infected cells. So, the next step is to verify the success of chimeric virion construction with plaque analysis. The diluted cell lysate containing wild-type SGIV and chimeric virions was inoculated into fresh GS culture until the appearance of plaques with GFP signal. The GFP-positive plaque is a niche with enriched chimeric virions, indicating the chimeric virus was generated successfully. The following steps aimed to concentrate and purify the chimeric virions. These GFP-positive plaques were picked up and the cell lysate was inoculated into GS cells for chimeric virions proliferation. To further enrich and purify chimeric virions, the GFP-positive cells were collected by cell sorting, and the sorted cells were lysed for next run of infection. After repeated plaque purification and cell sorting, the purity of chimeric virus in cell lysate increased until the *orf088* gene carried by wild-type SGIV was not detectable with PCR. In the following infection, the amount of VP088 encoded by wild-type SGIV was only enough to be assembled in a few portion of the progeny virions. The majority of progeny virions don’t have VP088 on capsid, and these virions are the desired product termed SGIVΔ88GFP. The deletion of VP088 didn’t totally block the life cycle of SGIVΔ88GFP. After the repeated plaque picking and cell sorting, the purity of SGIVΔ88GFP was maximized in cell lysate until the amount of VP088 protein encoded by wild-type SGIV was not detectable with Western blot analysis. The produced SGIVΔ88GFP was concentrated for further study. The other recombinant SGIV is SGIVp86GFP, which contains an inserted cassette expressing EGFP driven by the promoter of SGIV *orf086* as an IE gene (Fig. S2b). After verifying correct construction for gene deletion and addition by sequencing and Western blot analysis (Fig. S2c,d), both recombinant viruses were used for infection. They caused cytopathic effect (CPE) (Fig. S3a–c) and produced GFP signal in host cells at 3 days post infection (dpi) (Fig. S3b’,c’). We then compared the viral titer among SGIV, SGIVΔ88GFP and SGIVp86GFP. They were inoculated with HX1 cells at multiplicity of infection (MOI) of 0.1, and the titer was estimated for growth curve determination. It revealed that the titer of SGIVΔ88GFP dramatically reduced when compared with SGIV or SGIVp86GFP (Fig. 2d). Furthermore, we overexpressed VP088 in HX1 and examined the result of rescue to determine whether the reduced viral titer was indeed due to *orf088* deletion. Plasmid pVP88 encoding protein of VP088 was constructed (Fig. 3a; upper panel) and transfected into HX1 to produce stable transgenic cell line VP88HX1. As a control, cell line TgHX1 was developed by transfection of plasmid pcDNA3.1 into HX1. VP088 protein expression was verified by western blot analysis, where VP088 was detected as a single band protein of ~54 kDa in VP88HX1 but not in control cells (Fig. 3a; lower panel). VP088-expressing cell VP88HX1 was infected by SGIVΔ88GFP and this led to the significant rescue of SGIVΔ88GFP titer by VP088 expression in VP88HX1 cells (Fig. S4). Convincingly, VP088 deletion severely reduces the SGIV titer.

### VP088 does not alter viral gene expression and host response

The virus titer depends on the outcome of the interaction between virus and host. We examined the effect of VP088 overexpression or deletion on SGIV infection and host response. Transgenic VP088 expression did not alter properties of the host, as striking similarities were observed between VP88-HX1 and HX1 or TgHX1 in stem cell phenotype (Fig. S5a–c) and growth (Fig. S5d) analyzed as previously described^18^,^20^. The SGIV titer in VP88HX1 is not significantly higher than that in HX1 or TgHX1 (Fig. 3b), indicating that forced the VP088 expression doesn’t promote SGIV production. We then examined the effect of VP088 expression on host cell responsiveness to virus infection by determining the time-course expression of immune-responsive genes interferon (*ifn*) plus stat1 and cell death-associated genes *p53* plus *caspase*-3a (cas3a), as has previously been described^24^. A similar expression time course of viral gene *orf086*, *orf018* and *orf088* was detected between VP88-HX1 and HX1 or TgHX1 (Fig. 3c), suggesting that VP088 expression does not alter the host cell’s susceptibility and viral genes’ expression. There was also no difference in expression of immune-responsive host genes *ifn* plus *stat1* and cell death-associated genes *p53* plus cas3a (Fig. 3c). Taken together, VP088 overexpression has little effect on the expression profile of viral and cellular genes.

We also analyzed the effect of VP088 deletion on gene expression in HX1. On one hand, the *orf088* RNA was detected only in cells infected with SGIV or SGIVp86GFP as expected, whereas a similar time course and expression level of viral genes *orf086* and *orf018* were observed in HX1 cells infected with SGIV or its recombinant versions (Fig. 3d). On the other hand, *orf088* deletion had little effect on host genes’ expression, as no difference was detected between SGIVΔ88GFP and SGIVp86GFP in time course and expression level of selected cellular genes (Fig. 3d). For example, *ifn*, *p53* and *cas3a* exhibited upregulation at 6 hpi with either SGIVΔ88GFP or SGIVp86GFP but not with mock infection. Taken together, VP088 expression does not alter the expression of viral and cellular genes and host cell responsiveness to SGIV infection.

### VP088 deletion does not affect viral pathogenicity

**Figure 4:**
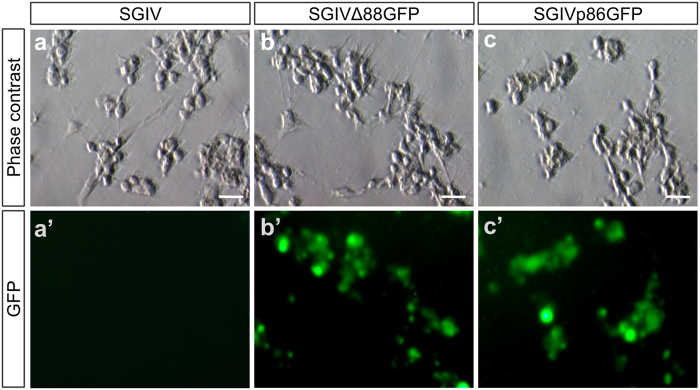
Pathogenicity of SGIV and its recombinant versions. HXI cells were infected with SGIV, SGIV∆88GFP and SGIVp86GFP at MOI of 1. CPE and fluorescent signal were observed with microscopy. (**a**,**a’)** HX1 cells at 72 hpi with SGIV. (**b**,**b’**) HX1 cells at 72 hpi with SGIV∆88GFP. (**c**,**c’**) HX1 cells at 72 hpi with SGIVp86GFP. CPE is seen in bright field phase contrast micrographs (**a–c**) and GFP expression is shown in fluorescent micrographs (**a’–c’**). Scale Bars, 10 μm.

The results presented so far have clearly demonstrated that SGIV neutralization, VP088 depletion or deletion leads to a dramatic reduction in viral titer (TCID_50_), a combined parameter of viral concentration, infectivity and pathogenicity. The pathogenicity here is defined as the ability of SGIV to cause CPE and/or cell death during its life cycle. We wanted to examine whether VP088 deletion will alter SGIV pathogenicity *in vitro*. For this, we compared the percentage of CPE after inoculated with SGIV, SGIVp86GFP or SGIVΔ88GFP at an identical MOI of 1 calculated from the titer (TCID_50_/ml) of virus stock (Table S1). As expected, SGIVΔ88GFP was not different from SGIV or SGIVp86GFP in the percentage of CPE production and CPE phenotype (Fig. 4). Therefore, a similar MOI leads to a similar percentage of CPE in host cells and VP088 deletion does not alter virus pathogenicity.

### VP088 deletion affects viral propagation

**Figure 5:**
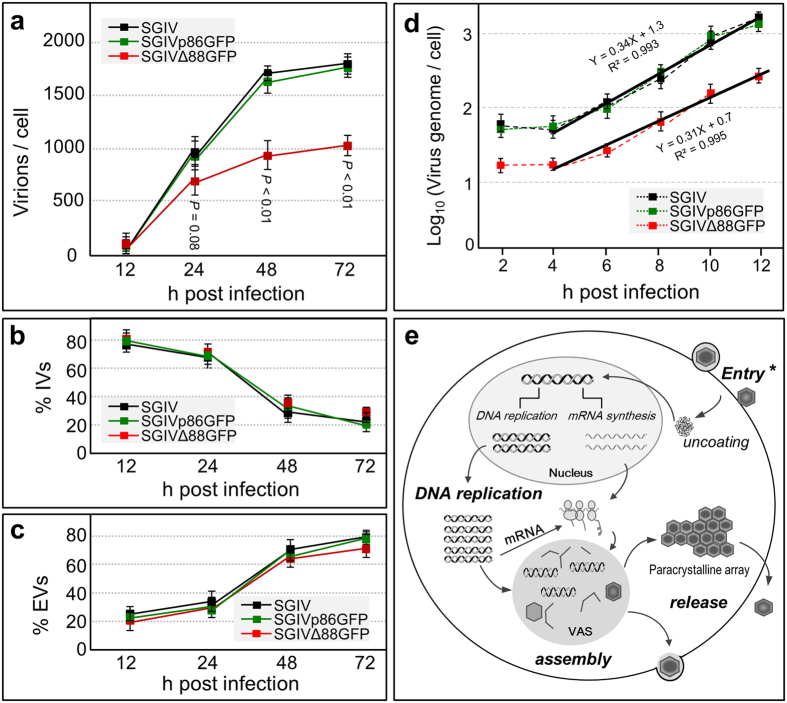
VP088 deletion affects SGIV infectivity. (**a–c**) ddPCR quantification of virions, showing the number of total virions (**a**) Ivs (**b**) and Evs (**c**) at 4 intervals post infection with SGIV, SGIVp86GFP and SGIVΔ88GFP at the same MOI. (**d**) The copy number of intracellular SGIV genome at early stages of infection. After infection with 10^2^ viral particles per cell in 6-well plates, DNA from HX1 cells at indicated hpi was extracted and quantified by the copy number of *orf072* as a representative gene of intracellular virus genome by ddPCR. (**e**) Schematic SGIV life cycle. Both naked and enveloped virions enter host cells, and release the viral DNA for replication and transcription in the nucleus. The replicated DNA exits from the nucleus and enters the cytoplasm for DNA replication and transcription. Viral protein concentrates in VAS for packaging into capsids. Newly assembled virions are accumulated as paracrystalline arrays in the cytoplasm or released from host cells. Asterisk depicts the SGIV infection step where VP088 plays its key role.

We then determined the number of total progeny virions during SGIV propagation. After inoculated with SGIV and its recombinant versions at MOI of 1, DNA isolated from purified virions and pelleted cells was loaded for ddPCR quantification respectively. In this study, *orf072* was used as a representative gene of the SGIV genome, and the cell number was determined by using *β*-actin as a reference. The copy number (CN) of *orf072* was calculated as CN_*orf072*_ = count_*orf072*_/count_*actin*_ to represent the number of virions produced per cell. SGIV virion propagation commenced already at 12 hpi, which became more evident until 24 hpi and continued to increase from 48 hpi to 72 hpi, as the number of total virions per cell was 65, 906, 1682 and 1831 at these 4 intervals (Fig. 5a). However, SGIVΔ88GFP produced similar numbers of virions at 12 and 24 hpi but significantly smaller numbers of virions at 48 and 72 hpi comparing to SGIV and SGIVp86GFP (Fig. 5a). This declined yield of SGIVΔ88GFP is consistent with our previously presented growth curves of SGIV and SGIVΔ88GFP determined by the TCID_50_ (Fig. 2d). During SGIV propagation, several steps are crucial for the yield of the virus including the replication of viral genome, release of assembled virus and the entry of progeny virus in the next run of infection. So, we subsequently examined which step(s) is/are affected in the life cycle of SGIVΔ88GFP after the deletion of VP088.

### VP088 deletion doesn’t affect virus release

SGIV virions can remain within the host as intracellular virions (IVs) or be released from the host as extracellular virions (EVs). To see whether VP088 played a role in the viral release, we determined the relative percentage of IVs and EVs. To this end, IVs and EVs were purified from intact cells and the cell culture supernatant respectively for copy number determination by ddPCR. The IV and EV percentage were calculated respectively comparing to the total virions (namely IVs plus EVs). After 24 hpi with virus, a steady decrease in IV percentage was observed (Fig. 5b), which was accompanied by a steady increase in EV percentage (Fig. 5c). For example, SGIV produced EVs at 23.1%, 30.4%, 70.3% and 77.9% at 12, 24, 48 and 72 hpi (Fig. 5c). These values are fully comparable to those obtained by infection with SGIVp86GFP and SGIVΔ88GFP (Fig. 5c), demonstrating the absence of any apparent difference between SGIVΔ88GFP and SGIV or SGIVp86GFP. Taken together, SGIV produces both IVs and EVs as early as 24 hpi and relatively more EVs at subsequent stages. Transgenic GFP expression or VP088 deletion does not affect SGIV release.

### VP088 deletion affects virus infectivity via inhibiting viral entry

The above observation that VP088 deletion does not significantly affect progeny virion release was based on the infection at the same MOI derived from TCID_50_ and it provoked us to further analyze the VP088 role in virus infectivity. We quantified virions number in previous virus stock used in CPE analysis and virus yield determination. The average virions number per TCID_50_ of SGIVΔ88GFP is 25.5, and the number of SGIV and SGIVp86GFP is 10.3 virions/TCID_50_ and 9.7 virions/TCID_50_ respectively (Table S1). Clearly, approximately 2.5 times of virions are needed for SGIVΔ88GFP to reach a similar percentage of CPE and progeny virion yield to those of SGIV or SGIVp86GFP, demonstrating that VP088 deletion reduced SGIV infectivity.

We then developed and utilized a new approach to precisely analyze infectivity by infecting host cells at one and the same number of input virions (NOI). Virus preparations were quantified for the absolute number of virions by ddPCR, and 10^8^ virions were infected to 10^6^ HX1 cells in 6-well plates (10^2^ virions/cell). DNA was isolated from carefully washed cells at various intervals and subjected to ddPCR determination. Shortly after infection at 2 hpi, the CN_*orf072*_ was 81 viral genomes/cell by SGIV infection which is fully comparable to 75 viral genomes/cell for SGIVp86GFP infection (Fig. 5d), suggesting that transgenic GFP expression does not alter SGIV infectivity. Notably, the CN_*orf072*_ reduced by up to 4-fold to 20 viral genomes/cell for SGIVΔ88GFP (Fig. 5d). These values remained unchanged until 4 hpi (Fig. 5d), indicating a similarity in the stability of delivered SGIV genome. Thus, VP088 deletion severely affects SGIV infectivity via inhibiting its entry without compromising the SGIV genome stability in the host.

Finally, we were interested in determining if VP088 was essential also for SGIV DNA replication, a key indicator of viral propagation. Since each infectious SGIV virion contains a single DNA and its replication correlates with viral propagation, we determined dynamic changes in viral genome copy number at critical hpi with SGIVΔ88GFP as well as SGIV or SGIVp86GFP on the basis of NOI of 10^2^/cell as described above. DNA was isolated from carefully washed HX1 cells at regular intervals up to 12 hpi, and copy numbers of viral genome and HX1 genome were determined by ddPCR. This revealed that the viral genome copy number increased steadily from 4 hpi to 12 hpi upon SGIV and SGIVp86GFP infection (Fig. 5d), which suggests continuous replication of the viral genome. An identical pattern was obtained also with SGIVΔ88GFP, with values at all time points examined being smaller than SGIV or SGIVp86GFP again by approximately 3.5-fold (Fig. 5d). Consequently, the slope of regression line remains unaltered by GFP expression and/or VP088 deletion. Thus, VP088 deletion primarily affects viral entry without altering the timing and efficiency of viral DNA replication and viral propagation.

## Discussion

Identification of key players and elucidation of their mechanistic roles in viral infectivity and pathogenicity are a precondition to innovate anti-viral strategies. In this study, we have identified VP088 as a key player in SGIV infection and unraveled its precise role in infectivity. This notion is supported by four independent lines of evidence. First, we have verified VP088 as the product of late gene *orf088* and as an envelope protein by its localization. Second, SGIV neutralization with the antibody against VP088, and VP088 knockdown or knockout leads to a remarkable decrease in SGIV titer, demonstrating an essential role for VP088 in SGIV infection. Third, VP088 overexpression or deletion does not alter viral and host gene expression profiles. Finally, we present convincing evidence that VP088 severely suppresses SGIV infection through inhibiting viral entry without altering viral pathogenicity, genome stability, replication and the release of assembled virus. Our results pinpoint the mechanism by which VP088 exerts its function: VP088 is dispensable for SGIV pathogenicity but indispensable for SGIV infectivity through controlling viral entry as the first step of infection cycle highlighted in the modified schematic figure^26^ (Fig. 5e). Our finding makes VP088 a strong candidate target for intervention of SGIV infection.

Usually, the viral titer is determined with TCID_50_, plaque formation unit or number of virions, and it has been frequently used in virus research^5^,^8^,^12^,^14^,^24^,^27^,^28^,^29^,^30^,^31^,^32^. The viral titer is measured depending on three major aspects, namely pathogenicity, infectivity and the number of infectious virions. To accurately quantify the number of virion makes it possible to discover the precise roles of key players in particular processes and aspects during infection. In this study, we have developed a new approach to exactly quantify the genome copy number throughout the infectious cycle, such as the number of virions in viral preparations from host cells as the output of infection or into host cells as the input of infection. This new approach enables a new infection procedure based on NOI and allows for quantifying the number of host cells, viral genome and/or virions for determining the aspect or step in which key players such as VP088 play a role.

## Conclusion

The viral life cycle consists of several major events such as entry, synthesis of macromolecular components, virus assembly and release. Here, we report a viral envelope protein VP088 is crucial for the infectivity of SGIV. Neutralization of SGIV with an antibody against VP088 could largely reduce infectivity of virions. Gene knockdown and knockout of *orf088* resulted in significant decrease of viral titer. Meanwhile, forced expression of VP088 in host could rescue *orf088* gene-deleted mutant. Furthermore, ddPCR quantification revealed knockout of VP088 significantly reduced the yield of virus after 3 days of infection and resulted in virions of low infectivity. Together, this work contributes greatly to identify VP088 as a crucial protein in SGIV infection and provides a potential target for viral disease control.

## Methods

### Cells and Virus

The medaka (*Oryzias latipes*) haploid ES cell line HX1 was maintained at 28 °C in medium ESM4 as previously described^33^,^34^. The grouper spleen cell line GS was maintained at 25 °C in medium Leibovitz L-15 as described^31^. SGIV (strain A3/12/98) was propagated in GS cells as described^5^. Briefly, SGIV was inoculated onto confluent GS cells at a multiplicity of infection (MOI) of ∼0.1. Upon the appearance of apparent cytopathic effect (CPE), cells and medium were collected, followed by three cycles of rapid freezing/thawing. Medium containing virus was centrifuged at 12,000 g (Eppendorf) for 30 min at 4 °C, the supernatant was centrifuged at 200,000 g (Beckman) for 1 h at 4 °C to concentrate virus, and the pellet was resuspended in L-15 and stored at −80 °C until use.

### Plasmids

See Supplementary methods.

### Antibody

A customized monoclonal antibody against VP088 (αVP088) was developed in mice (Abmart). In brief, peptide DAKAEVGEAA within the VP088 C-terminus was synthesized and conjugated to keyhole limpet hemocyanin as an immunogen to mouse. The hybridoma was generated and titers of ascites containing antibody against synthetic peptide were tested by ELISA.

### Protein expression and western blot analysis

Plasmid pET88C was transformed into *E.coli* BL21 (DE3) by heat-shock. Protein expression was induced with 0.5 mM IPTG at 18 °C overnight. Soluble rVP88 with 6 × his tag was purified with Ni-NTA beads according to instruction (Qiagen, USA) and stored at −20 °C until use. Recombinant protein rVP88 and crude proteins from SGIV-infected HX1 cells were loaded for western blot analysis by using αVP088 and antibody to β-Actin (clone AC-74, Sigma) as the primary antibody respectively.

### Neutralization assay

Purified SGIV (10^5^ TCID_50_/ml) was incubated with αVP088 at final concentrations ranged from 0.2 to 8 μg IgG/ml for 30 min at 25 °C. Meanwhile, the same amount of SGIV was incubated with irrelevant antibody anti-GST IgG (8 μg/ml, Abcam, ab21070) as a control. After incubation, 100 μl of a mixture containing SGIV and antibody was added into each well with 1 × 10^5^ GS cells inside. After inoculation for 2 h at 25 °C, the inoculum was removed and 1 ml of culture medium was added. The infected cells together with supernatant were collected at regular intervals and stored at −80 °C until titer determination by using 50% tissue culture infectious dose (TCID_50_) in GS cells at 72 h post infection (hpi).

### Morpholino gene knockdown

Three antisense morpholino oligos (MOs) used in this study were purchased from Gene Tools (Oregon, USA). MO88 (TTACGGATTGCGCTGCGCCCATTTT) targets *orf088.* MO72 (GTACAAGTCATTGTTGCTGTTTTTT) targets *orf072* encoding the SGIV major capsid protein^6^. MOctr (CCTCTTACCTCAGTTACAATTTATA) is a general control morpholino oligo that has no target gene in HX1 and SGIV. Briefly, HX1 cells at 70% confluence in 6-well plates were transfected with 10 μM of MOctr, MO72 or MO88 by using the Endo-Porter reagent (Gene Tools, Oregon, USA). At 24 h post transfection, cells were inoculated with SGIV at MOI of 0.1 and virus titers were determined at 72 hpi.

### Generation of recombinant viruses

Plasmid pBKO88GFP was transfected into GS cells before inoculation of SGIV, and *orf088* knockout recombinant SGIV was generated by homologous recombination in host. In detail, 2 μg of plasmid pBKO88GFP and 8 μl of DNAfectin were mixed in 200 μl of serum-free L-15 medium. After incubation at room temperature for 20 min, the transfection mixture was added dropwise to cells in 6-well plate containing 2 ml of serum-free medium. After incubation for 6 h at 25 °C, transfection mixture was removed and cells were subsequently cultured in complete medium for 12 h. Subsequently, the cells were infected with SGIV at MOI of 0.1 and monitored under fluorescent microscopy routinely until appearance of green fluorescence signal and CPE in host. Then, cells were collected with centrifugation for 10 min at 3,000 g. Excess supernatant was removed and cell pellets were suspended with 300 μl of fresh medium. The cell suspension was frozen-thaw repetitively for 3 times and serially diluted before inoculation with GS cells cultured in 10-cm petri dish. After 72 hpi, viral plaques with green fluorescent were picked up with 200-μl tips, and the cells lysate was inoculated to GS cells cultured in 48-well plate for recombinant virus propagation. To further purify the recombinant virus, green fluorescent signal positive cells were sorted by fluorescence-activated cell sorting (FACS) with BD FACSAria flow cytometer. After several runs of plaque purification and sorting, purified recombinant viruses was tittered and stored at −80 °C for further experiment. Meanwhile, the *egfp* gene knock-in virus SGIVp86GFP was generated following the same procedure with pBKIGFP.

To verify the recombinant virus with PCR, purified viruses of SGIVΔ88GFP and SGIVp86GFP was inoculated into GS cells (MOI = 0.1) separately until CPE was apparent. Then, cells were collected with centrifugation for total DNA extraction as previously described^24^. In brief, GS cells were incubated in lysis buffer [10 mM Tris/HCl (pH 8.0), 1 mM EDTA, 1% SDS, proteinase K (100 mg/ ml)] at 50 °C for 3 h. After phenol:chloroform extraction, DNA was precipitated by adding 2 volumes of ethanol at −20 °C overnight and centrifuged at 12,000 g for 15 min. The DNA pellet was resuspended in distilled water with RNase A (50 μg/ml). Diagnostic PCRs were run at 30 cycles (95 °C for 30 sec, 56 °C for 20 sec and 72 °C for 4 min) in a 20-μl volume reaction containing 250 ng of DNA. Primers of upEcoR plus downKpn were used to amplify target fragment from SGIVΔ88GFP; LFEcoF plus downKpn for fragment from SGIVp86GFP and GFPHindF plus GFPXho for the inserted e*gfp* gene. PCR products were electrophoresed in 0.8% agarose gel and recovered with gel extraction kit (Qiagen) for sequencing.

### Generation of stable transfected cell lines

See Supplementary methods.

### RT-PCR and ddPCR

The RT-PCR analyses were performed as described^17^,^24^. PCR was run for 25 cycles (*β-actin* as an internal control, 95 °C for 30 sec, 60 °C for 20 sec and 72 °C for 1 min) or 35 cycles (other genes) in a 20-μl volume reaction containing 10 ng of cDNA. PCR products were loaded for electrophoresis on 2% agarose gels. Primers are listed in Supplementary Table S2.

Droplet digital PCR (ddPCR) is superior to real-time PCR for DNA quantification due to its excellent precision, sensitivity and reproducibility^35^,^36^. We used ddPCR to quantify the viral DNA or virions by determining the absolute copy number of viral genes. To assess the virion yield in each infection, HX1 cells were inoculated with SGIV and its recombinant versions at an identical MOI calculated from the titer, and progeny virus particles in the host cells and supernatant were individually collected for analysis. Then, the viral DNA extracted from virus is loaded for ddPCR quantification. In detail, HX1 cell (2 × 10^6^) were inoculated with SGIV, SGIVp86GFP or SGIVΔ88GFP at MOI of 1 respectively. After infection for 2 h, access viruses were removed by several runs of washing with PBS. At designed hpi, virus particles in supernatant and host cell were collected respectively. Medium containing virus was centrifuged at 12,000 g (Eppendorf) for 30 min at 4 °C to remove the cell debris. Then, the supernatant was transferred to a new tube and incubated with the additional DNase I (NEB, 5 units/ml) for 20 min at 37 °C. After digestion, the DNase I was inactivated at 75 °C for 10 min. After eliminating the DNA contamination, the solution was loaded for ultracentrifugation at 200,000 g (Beckman) for 1 h at 4 °C to concentrate the virus. The pellet was washed with PBS for 3 times by ultracentrifugation. Then, the purified virus particles were incubated with lysis buffer and the viral DNA was extracted with the mentioned DNA extraction method for cells. Subsequently, the viral DNA was loaded for ddPCR analysis, and viral gene copy number was quantified by taking *orf072* as the target gene. Meanwhile, the *β-actin* gene in host was quantified as a reference as previously described^37^. In brief, ddPCR assay mixtures (25 μl/reaction) containing 12.5 μl 2X EvaGreen Super Mix, 40–60 ng *Hind*III digested genomic DNA, and 100 nM primers (Table S2) were loaded onto droplet generator (Bio-Rad) for droplets generation. The droplets were subjected to PCR amplification following the condition: 95 °C for 5 min; 40 cycles of 95 °C for 30 s and 60 °C for 1 min; and 3 final steps at 4 °C for 5 min, 90 °C for 5 min, and hold at 4 °C to enhance dye stabilization. Droplet counting was subsequently performed on QX100 Droplet Reader (Bio-Rad) and result was analyzed with Quantasoft software (Bio-Rad).

Meanwhile, ddPCR was also used to assess infectivity of SGIV and its recombinant versions. In brief, virion number of each stock containing SGIV, SGIVp86GFP or SGIVΔ88GFP was quantified with ddPCR by taking *orf072* as the target gene. Then, an equal number of SGIV and recombinant virions (10^8^ virions) were inoculated with 10^6^ cells respectively for 2 hours. The access virions were removed by 3 times of washing with PBS, and the infected cells were trypsinized and collected at designed hpi for total DNA extraction. Gene copy number of *orf072* and *β-actin* in each infection was quantified with ddPCR for virus infectivity assessment.

### Immunostaining and Microscopy

See Supplementary methods.

### Statistical analysis

The Dunnett’s test was conducted by using GraphPad Prism v4.0. Data were presented as means ± S.D. P values were calculated by using the Student’s *t*-test and *P* < 0.05 was considered as significantly different as described.

## Additional Information

**How to cite this article**: Yuan, Y. *et al*. Singapore grouper iridovirus protein VP088 is essential for viral infectivity. *Sci. Rep.* **6**, 31170; doi: 10.1038/srep31170 (2016).

## Supplementary Material

Supplementary Information
