# Supplementary material for: Singapore grouper iridovirus protein VP088 is essential for viral infectivity
Source: Sci Rep. 2016 Aug 8;6:31170. doi: 10.1038/srep31170 (PMC4976331; doi:10.1038/srep31170)

**Singapore grouper iridovirus protein VP088 is essential for viral infectivity**

**Yongming Yuan, Yunzhi Wang, Qizhi Liu, Feng Zhu, Yunhan Hong***

Department of Biological Sciences, National University of Singapore, Science Drive 4, Singapore 117543, Singapore

*Correspondent author: Professor Yunhan Hong

Department of Biological Sciences

National University of Singapore

Science Drive 4

Singapore 117543

E-mail: [dbshyh@nus.edu.sg](mailto:dbshyh@nus.edu.sg)

Tel: +65-65162915; Fax: **+65-67792486**

Supplementary files list:

1. **Supplementary methods**
2. **Supplementary tables**

**Table S1.** Viral titer, infectivity and pathogenicity

**Table S2.** Genes and primers used for RT-PCR and ddPCR analyses

1. **Supplementary figures**

**Figure S1.** VP088 Knockdown

**Figure S2.** Generation of recombinant virus

**Figure S3.** Infection of SGIV and recombinant variants

**Figure S4.** Rescue of VP088-deletion phenotype

**Figure S5.** Effect of transgenic VP088 expression on host cells

**Supplementary Methods**

**Plasmids**

Plasmid pET88C was constructed for prokaryotic expression of rVP88, the C-terminal part of VP088. Briefly, the *orf088* DNA fragment encoding a 248 C-terminal amino acids was PCR-amplifieded with primers 88cEco (aagaattcgccgtagaaaacg, underlined letters depicting introduced restriction site) and orf088Hind (gcaagcttctttgcagcttc) and inserted in frame between *EcoR*I and *Hind*III sites in pET32a.

Plasmid pVP88 expressing the full-length of VP088 was constructed by inserting *orf088* into vector of pcDNA3.1. Briefly, the *orf088* coding sequence (CDS) was amplified by using primers orf088Eco (aagaattcaccATGGGCGCAGCGC) plus orf088Hindt (gcaagcttTTActttgcagcttc) from the SGIV genome. The PCR product was digested with *EcoR*I plus *Hind*III and released fragment was combined with *EcoR*I-*Hind*III double-digested pcDNA3.1 for ligation. Plasmid pBKO88GFP was constructed to knock out *orf088* by homologous recombination. It contains gene *egfp* between the flanking regions of *orf088* (Fig. S2a). Briefly, *egfp* was PCR-amplified from pEGFP-N1 (Clontech) with primers GFPBam (aggatccATGGTGAGCAAGGGCGAG) and GFPXho (actcgagTTACTTGTACAGCTCG). The left and right flanking regions of *orf088* (nucleotides 82021 to 82666 and 84158 to 84900) were PCR-amplified from the SGIV genome with primers upEcoR (agaattcacaacagtactcacag) plus 88UFBamR (cggatccttttaaagatcgttttc) and downxho (actcgagcgacattgtaaatttc) plus downKpn (aggtaccttagattcttaaattat), respectively. The amplified DNA fragments were cloned into pBluescript II KS (+) between *EcoR*I and *Kpn*I sites.

Plasmid pBKIGFP was constructed to generate knock-in virus by inserting e*gfp* between *orf087* and *orf088* in the SGIV genome through homologous recombination. To drive GFP expression, the inserted marker cassette contains a 180 bp upstream region from transcription start point of SGIV *orf086* (Fig. S2b). Briefly, a 180 bp fragment termed 086prom was PCR-amplified with primers Pro86NheF (agctagcagtgccccaaatgcgg) plus Pro86HindR (aaagcttggtgtttggtagtgtttac) and inserted upstream of e*gfp* gene PCR-amplified with primers GFPHindF (caagcttATGGTGAGCAAGGGCGAG) and GFPXho. Two DNA fragments (nucleotides 82021 to 82346 and 82347 to 82655) which act as the flanking regions were PCR-amplified with primers LFEcoF (agaattcatttcttaacttttttgg) plus LFNheR (cgctagctgataaaacttccctaac) and RFXhoF (actcgagtcgggggaccctatgc) plus downKpn. These fragments were ligated into pBluescript II KS (+) between *EcoR*I and *Kpn*I sites. Correct cloning was confirmed by test digestion and sequencing. Plasmid DNA used for cell transfection was prepared by using the Midiprep kit (Qiagen, Valencia, CA, USA).

**Generation of stable transfected cell line**

HX1 cells at 70% confluence were transfected with DNAfectin reagent (Applied Biological Materials, Richmond, BC, Canada) in 6-well plates. In brief, 2 µg of plasmid pVP88 and 8 µl of DNAfectin reagent were mixed in 200 µl of pure DMEM (Dulbecco's modified eagle medium). After incubation at room temperature for 20 min, the transfection mixture was added dropwise to cells in a well of 6-well plate containing 2 ml of DMEM. After incubation for 6 h at 28°C, the cells were cultured in ESM4 for 48 h. The transfected cells were subcultured at serious dilution into 10-cm gelatin coated petri dish containing 0.5 mg/ml G418 in ESM4 and cultured till the formation of individual colonies, with medium changing every 5-7 days. Each colony was picked with 200-µl tips into 96-well plates. To screen out the colony expressing VP088, cells in each well were sampled for western blot analysis and the positive colony was further expanded into cell line of VP88-HX1. Another cell line of TgHX1 was expanded from drug resistant colony derived from cells transferred with pcNDA3.1. The cell growth kinetics was analyzed as described . Briefly, 10^5^ of cells were seeded into 6-well plate and counted in triplicates every 24 h until 8 days of culture.

**Immunostaining and Microscopy**

For confocal microscopic observation, samples were immunostained before observation. In brief, HX1 cells cultured at glass coverslips at 0, 24, 36 and 60 hpi with SGIV were washed with PBS and fixed with 4% paraformaldehyde for 4 h at 4°C. After washing, samples were permeabilised with 0.2% Triton X-100 for 10 min. Then, cells were blocked with PBS containing 4% bovine serum albumin (BSA) and 0.05% Tween-20 for 20 min. Primary antibody **α**VP088 was applied to sample with dilution of 1:200 and incubated for 3 h at room temperature. After thoroughly wash with PBS, Alexa 488 conjugated goat anti-mouse antibody (Invitrogen, USA) was added at a dilution of 1:500 and incubated for another 2 h. To visualize the F-actin of host, cells were incubated with Alexa Fluor 568 Phalloidin (200 units/ml, Invitrogen, USA) for 30 min. To stain nucleus and concentrated viral DNA in host cells, 4', 6'-diamidino-2-phenylindole (DAPI) was added at the concentration of 0.5 μg/ml. Confocal microscopy (UltraView VoX, PerkinElmer) was used to analyze the sections with 40X water immersed lens (numerical aperture = 1.15). Software of Volocity 6.2.1 was set at 3 channels for sequentially recording modes with laser lines at 405 nm, 488 nm and 561 nm. Images were exported with the same software. Observation and photography of cells in plates were performed on Zeiss Axiovert invert microscope with a Zeiss AxioCam M5Rc digital camera (Zeiss Corp., Germany).

To locate the VP088 protein, the immunoelectron microscopy (IEM) was performed. In detail, the SGIV-infected HX1 cells at 60 hpi were fixed with 4% paraformaldehyde and 0.1% glutaraldehyde overnight at 4°C. The fixed cells were washed with PBS and dehydrated in ethanol (30, 50, 70, 90, 100%; 10 minutes each) and infiltrated with LR white at gradient concentration. The resin was polymerized for 48 hours at 4°C under UV light. Polymerized blocks were sectioned with a diamond knife (Diatome) on an ultra-microtome (Leica EM FCS). Ultrathin sections of 200-nm thickness were loaded onto 100-mesh nickel grids. The sections were blocked by incubation in PBS containing 4% bovine serum albumin and 0.05% Tween-20 for 20 min. The blocked sections were incubated with primary antibody **α**VP088 or an irrelevant antibody against GFP protein (**α**GFP, **ab1218; Abcam**) as a parallel control at a 1:100 dilution for 3 h and washed thoroughly with PBS, followed by incubation for 3 h with secondary antibodies conjugated with 1.4-nm nanogold (Cat.2001, Nanoprobe). After washing in distilled water with 3 changes, the sections were subjected to gold-enlargement procedure according to supplier’s instruction (Cat.2113, Nanoprobe) to an appropriate size for EM analysis. Ultrathin sections were examined on a 120 kV transmission EM (FEI Tecnai T12) and photographed on a Gatan 4K × 4K CCD camera (Gatan Corporation).

**Supplementary Tables**

**Table S1. Viral titer, infectivity** and pathogenicity

| Virus | Titer (×10^7^) | | Infection (MOI) | | Progeny virions/cell* | |
| --- | --- | --- | --- | --- | --- | --- |
|  | TCID_50_/ml | Virions/ml* | TCID_50_/cell | Virions/cell | Total virions | Extracellular, n (%) |
| SGIV | 1 | 10.3±0.9 | 1 | 10.3±0.9 | 1831±83 | 1426±72 (77.9) |
| SGIVp86GFP | 1 | 9.7±0.6 | 1 | 9.7±0.6 | 1809±86 | 1432±61 (79.1) |
| SGIV∆88GFP | 1 | 25.5±0.7 | 1 | 25.5±0.7 | 1105±78 | 803±89 (72.7) |
| *, Number of virions was determined with ddPCR-quantified SGIV genome copy number | | | | | | |

**Table S2. Genes and primers used for RT-PCR and ddPCR analyses**

| Gene | Accession number | Primer | Sequences (5’ to 3’) | Size (bp) |
| --- | --- | --- | --- | --- |
| *orf072* | AAS18087.1 | ORF072F | AACGCATAGGTTACGACAAC | 268 |
|  |  | ORF072R | GTAGCAGAGATAGGAATCAC |  |
| *orf086* | AAS18101.1 | ORF086F | GCCATTCAACTGACACTTTG | 459 |
|  |  | ORF086R | AACCCCTTGGATGGTAAATTC |  |
| *orf088* | AAS18103.1 | ORF088F | ATGGGCGCAGCGCAATC | 520 |
|  |  | ORF088R | CGATGTCGCTGAGCTG |  |
| *interferon* | XP_004085828.1 | InterferonF | CCTTCACGGTCCAGGTTC | 248 |
|  |  | InterferonR | GATGGTCTCCCAGGCTTC |  |
| *stat1* | ENSORLG00000000162 | STAT1F | CACTCCGTTGAGCCGTAC | 379 |
|  |  | STAT1R | CAAACCCTCCAAAGTCCAT |  |
| *p53* | AAG48557.1 | P53F | CTGTGGAGCACCGAAGCCATC | 551 |
|  |  | P53R | AAGACGCTCTGTCTTCCTGTGA |  |
| *cas3a* | NP_001098140.1 | Cas 3AF | CCGACCCTTACCGCTAC | 429 |
|  |  | Cas 3AR | CGTGCTCTATGCCCTCAT |  |
| *β-actin*  (RT-PCR) | S74868.1 | ActinF | TTCAACAGCCCTGCCATGTA | 650 |
|  |  | ActinR | cctccaatccagacagagta |  |
| *β-actin*  (dd-PCR) | S74868.1 | ddctinF | GCTTTATGCCAGCAACGACT | 157 |
|  |  | ddctinR | GAGGGACTTTAGACTATCGG |  |

**Supplementary Figures**


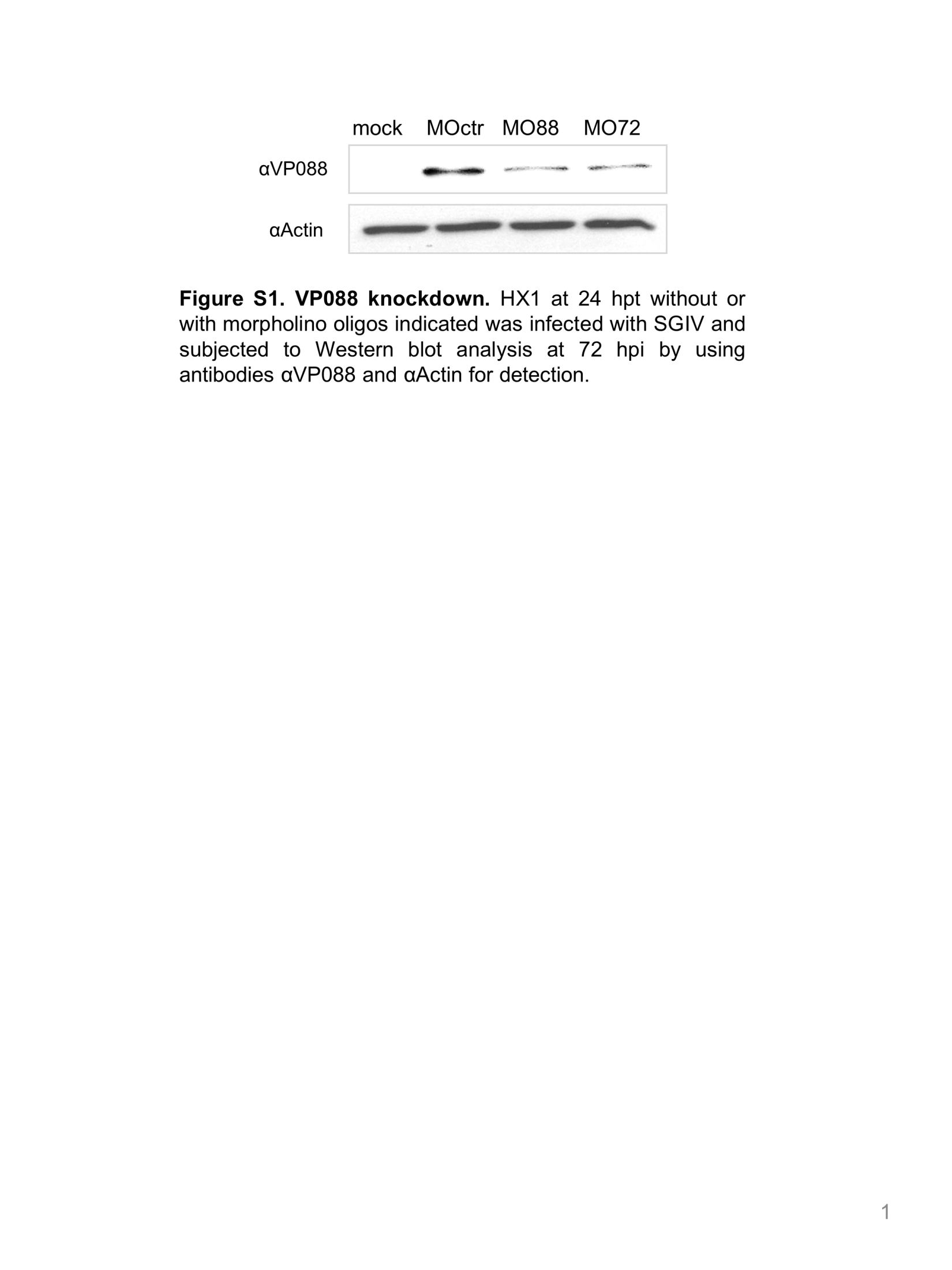


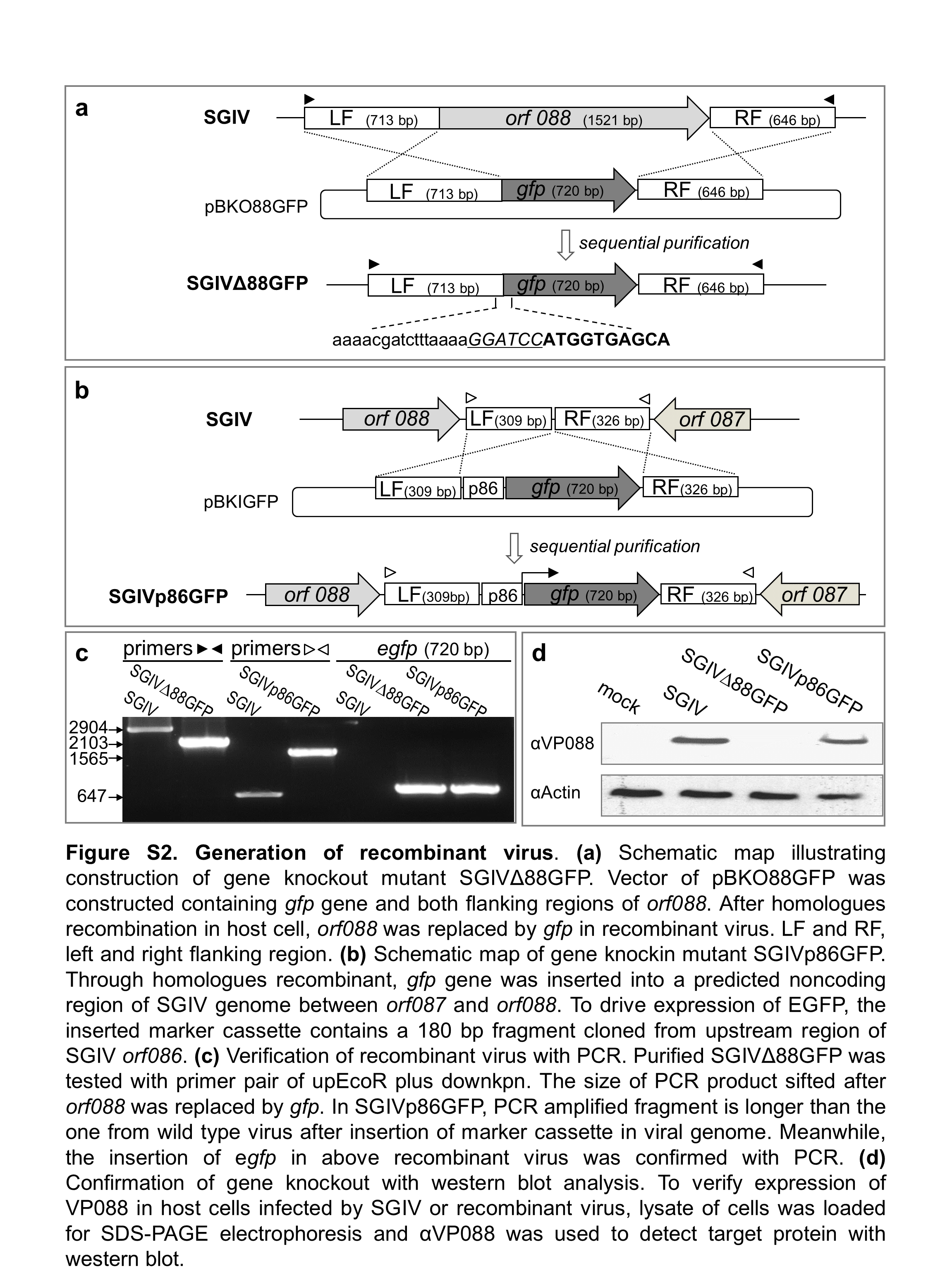


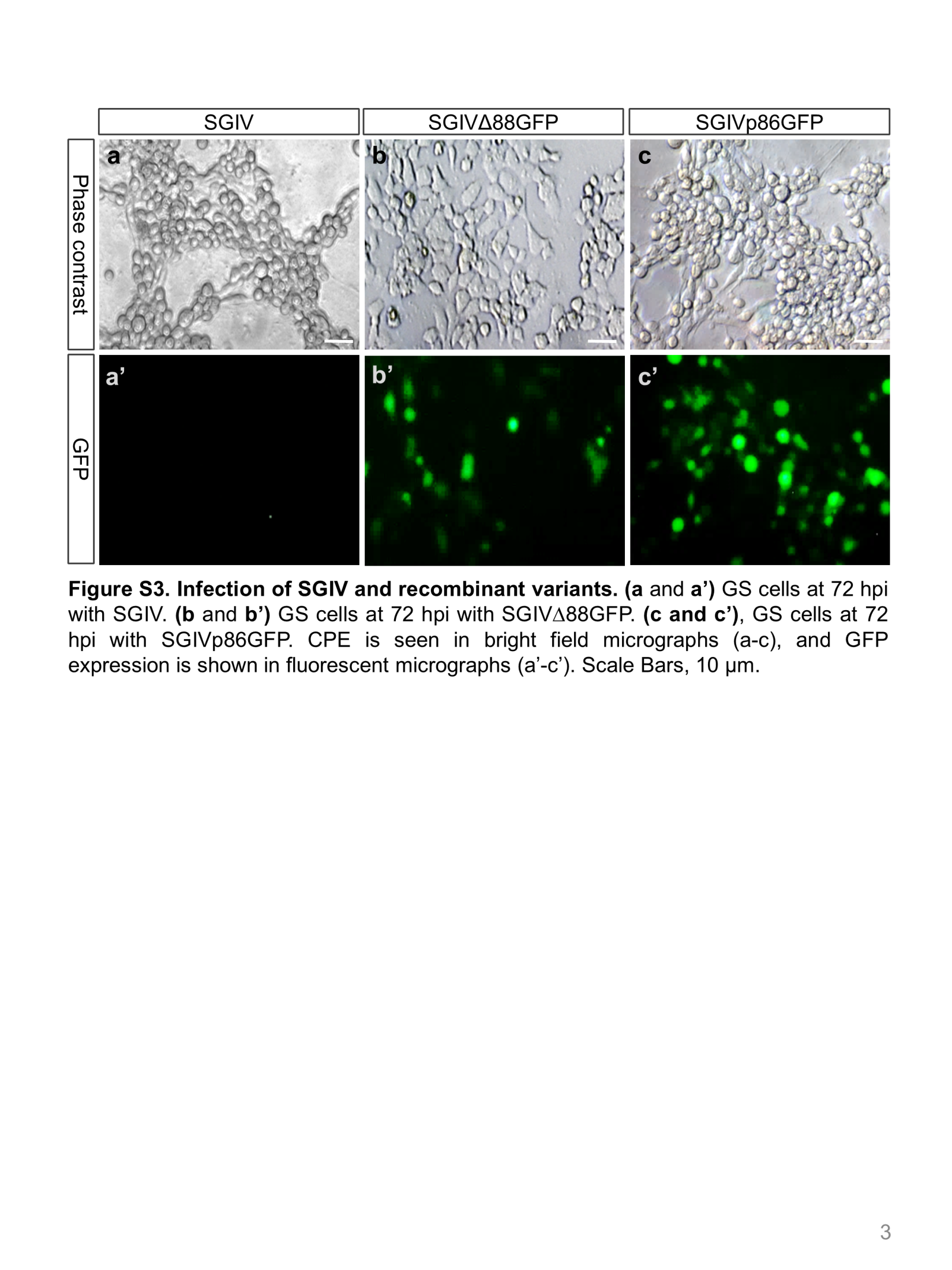


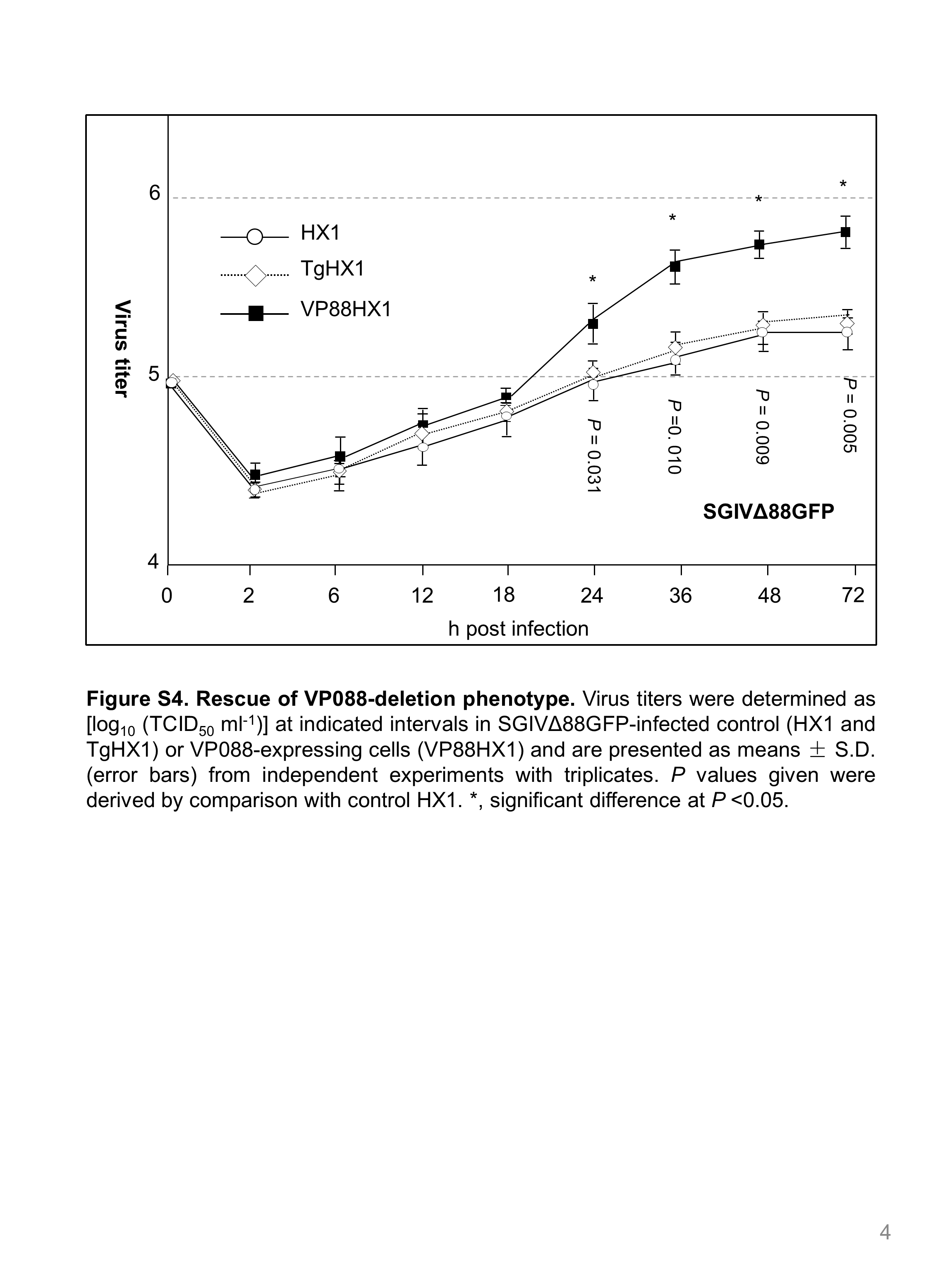


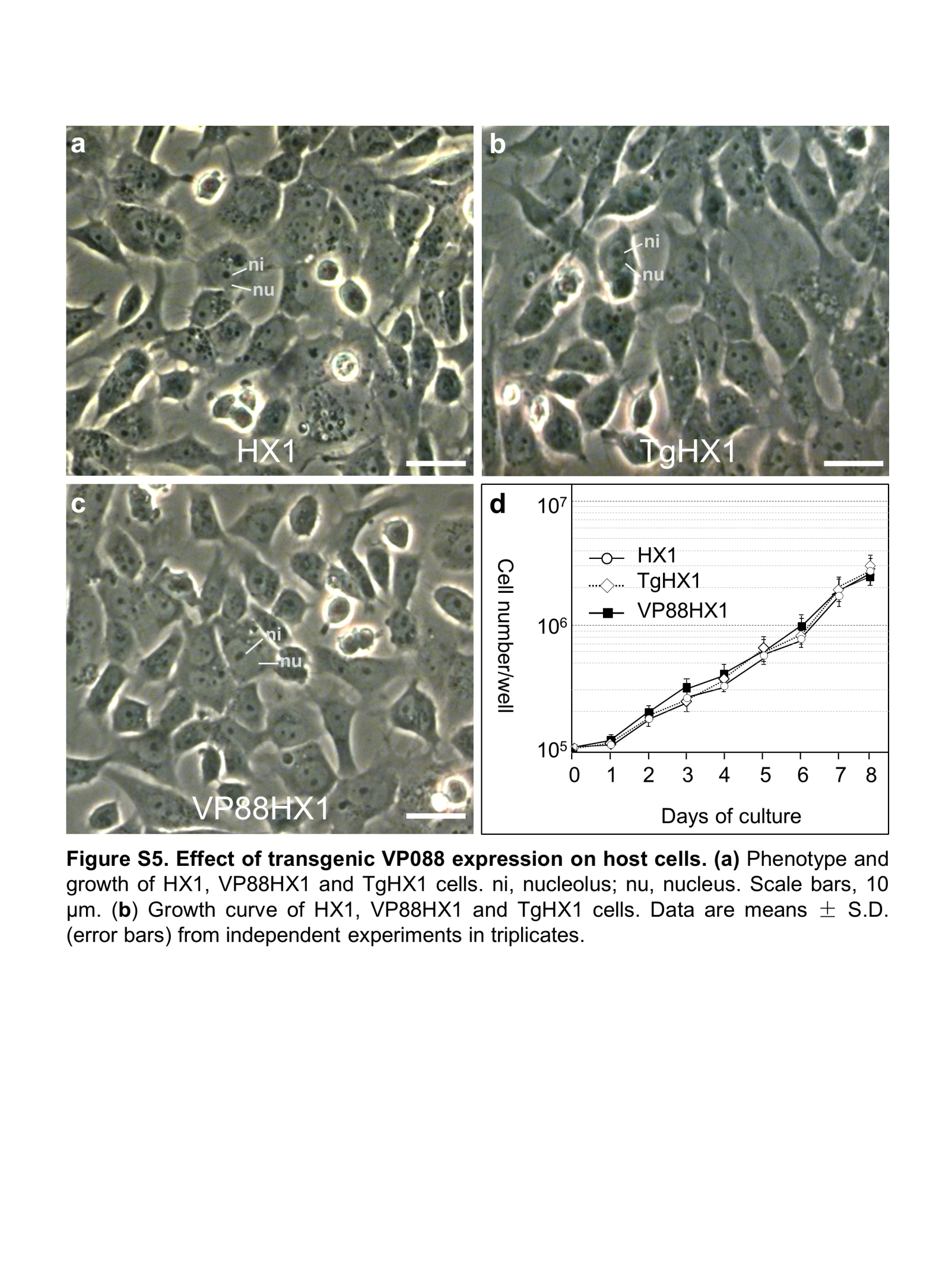

Supplement: Supplementary Information [file srep31170-s1.doc]
